# Redox Factor-1 Activates Endothelial SIRTUIN1 through Reduction of Conserved Cysteine Sulfhydryls in Its Deacetylase Domain

**DOI:** 10.1371/journal.pone.0065415

**Published:** 2013-06-03

**Authors:** Saet-Byel Jung, Cuk-Seong Kim, Young-Rae Kim, Asma Naqvi, Tohru Yamamori, Santosh Kumar, Ajay Kumar, Kaikobad Irani

**Affiliations:** Division of Cardiology, Department of Medicine, University of Pittsburgh, Pittsburgh, Pennsylvania, United States of America; Goethe University, Germany

## Abstract

Apurinic/Apyrmidinic Endonuclease 1/Redox Factor-1 (APE1/Ref-1) is a reductant which is important for vascular homeostasis. SIRTUIN1 (SIRT1) is a lysine deacetylase that also promotes endothelium-dependent vasorelaxation. We asked if APE1/Ref-1 governs the redox state and activity of SIRT1, and whether SIRT1 mediates the effect of APE1/Ref-1 on endothelium-dependent vascular function. APE1/Ref-1 maintains sulfhydryl (thiol) groups of cysteine residues in SIRT1 in the reduced form and promotes endothelial SIRT1 activity. APE1/Ref-1 stimulates SIRT1 activity by targeting highly conserved vicinal thiols 371 and 374 which form a zinc tetra-thiolate motif in the deacetylase domain of SIRT1. Cysteine residues in the N-terminal redox domain of APE1/Ref-1 are essential for reducing SIRT1 and stimulating its activity. APE1/Ref-1 protects endothelial SIRT1 from hydrogen peroxide-induced oxidation of sulfhydryls and from inactivation. APE1/Ref-1 also promotes lysine deacetylation of the SIRT1 target endothelial nitric oxide synthase (eNOS). SIRT1 mutated at cysteines 371 and 374, which renders it non-reducible by APE1/Ref-1, prevents lysine deacetylation of eNOS by APE1/Ref-1. SIRT1 free thiol (reduced sulfhydryl) content and deacetylase activity are diminished in all examined tissues of APE1/Ref-1^+/−^ mice, including the vasculature. Overexpression of SIRT1 in aortas of APE1/Ref-1^+/−^ mice restores endothelium-dependent vasorelaxation and bioavailable nitric oxide (NO) to levels similar to those observed in wild-type mice. Thus, APE1/Ref-1, by maintaining functionally important cysteine sulfhydryls in SIRT1 in the reduced form, promotes endothelial SIRT1 activity. This reductive activation of endothelial SIRT1 by APE1/Ref-1 mediates the effect of APE1/Ref-1 on eNOS acetylation, promoting endothelium-derived NO and endothelium-dependent vasorelaxation.

## Introduction

The NAD^+^-dependent SIRT1 protein deacetylase, the closest mammalian ortholog to yeast Silent Information Regulator-2 (Sir2), has myriad roles in different organs, and has gained enormous attention as a mediator of changes in mammalian metabolism and physiology [1]. SIRT1 is regulated at the transcriptional and post-translational levels. It is post-translationally modified by sumoylation [2], and phosphorylation [3]. In addition, cysteine residues in SIRT1 are S-glutathiolated [4], nitrosylated [5], and alkylated [6]. Specific cysteine residues in the catalytic domain of SIRT1 form a zinc tetra-thiolate center, and are highly conserved from yeast to mammals [7]. Mutation of these conserved cysteines in yeast Sir2 abolishes its function [8], as does chelation of zinc [7], [9]. The importance of these specific thiol residues to Sir2 activity, as well as the role of thiol modifications in general on SIRT1 activity, suggests the presence of endogenous reductants that regulate the redox status of cysteine residues, and activity, of SIRT1.

Redox factor-1 (Ref-1) is a ubiquitously expressed protein, mainly nuclear in localization, which has multiple functions [10]. Also known as Apurinic/Apyrmidinic Endonuclease-1 (APE1), it is the rate limiting enzyme in the mammalian base excision repair pathway [11]. As a cellular reductant, APE1/Ref-1 targets many transcription factors, maintaining functionally important cysteine residues in these transcription factors in the reduced form [12]. Reduction of these transcription factors by APE1/Ref-1 promotes their binding to DNA or transcriptional co-activators. In the endothelium, APE1/Ref-1 is an important mediator of endothelial homeostasis: it stimulates nitric oxide (NO) production, and promotes endothelium-dependent vasodilation [13].

SIRT1 binds to and deacetylates APE1/Ref-1, stimulating APE1/Ref-1-mediated DNA repair [14]. Recognizing the importance of cysteine modification in regulating SIRT1 activity, and the role of APE1/Ref-1 as a cellular reductant, we hypothesized that the functional impact of the relationship between SIRT1 and APE1/Ref-1 may be mutual - that APE1/Ref-1 also has an important role in regulating SIRT1 activity. In this work we asked if APE1/Ref-1, by reducing thiol (sulfhydryl) moieties of cysteine residues in SIRT1, is important for SIRT1 activity in endothelial cells, and whether SIRT1 mediates APE1/Ref-1-stimulated endothelium-dependent vasorelaxation.

## Materials and Methods

### Cell Culture, Plasmids, siRNA, and Transfections

Human embryonic kidney (HEK 293) cells were purchased from American Type Culture Collection and cultured in DMEM supplemented with 10% FBS and antibiotics. The SIRT1 (C371S/C374S) and APE1/Ref-1 (C65A/C93A) mutants were created in wild-type cDNA templates (generously provided by T. Kouzarides and T. Finkel, respectively) by site-directed mutagenesis using the QuickChange kit (Stratagene). All mutations were verified by sequencing. Small interfering RNA for human APE1/Ref-1 (5′-CCU CAA UGU GGC ACA UGA AGA AAU U-3′), and the appropriate scrambled (scr) control siRNA were purchased from InVitrogen. Cells were transfected with plasmids and siRNA using Lipofectamine2000 (InVitrogen) per recommendations of the manufacturer. All other reagents were purchased from Sigma.

### Immunoblotting and Immunoprecipitations

Antibodies against SIRT1 (Santa Cruz Biotechnology), APE1/Ref-1 (Santa Cruz Biotechnology), GST (Santa Cruz Biotechnology), Myc epitope (Santa Cruz Biotechnology), eNOS (Santa Cruz Biotechnology) and Acetylated-Lysine (Cell Signaling) were purchased. Immunoprecipitations of SIRT1and myc –SIRT1 were carried out with overnight incubation of 2 µg of antibodies and 1 mg of nuclear extract (Nuclear extraction kit, Cayman) from cell lysate or tissue homogenate, followed by 30 µL of protein A–sepharose slurry (Amersham) for 4 hours. Immunoprecipitation of eNOS and acetyl-lysine was similarly carried out using whole cell lysate. After washing, immunoprecipitates were boiled in SDS-PAGE loading buffer, subjected to SDS-PAGE, transferred to nitrocellulose filter, and probed with the specified primary antibody and the appropriate peroxidase-conjugated secondary antibody (Santa Cruz Biotech). Chemiluminescent signal was developed using Super Signal West Pico or Femto substrate (Pierce), and blots imaged with a Gel Doc 2000 Chemi Doc system (BioRad).

### Immunohistochemistry

De-parafinnized mouse aortic rings sections were permeabilized and processed using a Vectastain Universal Quick Kit (Vector Laboratories, PK-8800). Primary antibody to Acetylated-p53 (Cell Signaling) was used at a 1∶50 dilution followed by biotinylated secondary antibody, streptavidin peroxidase solution, DAB peroxidase substrate, and hematoxylin counterstain. Sections were digitally imaged with a Zeiss Axiovert 200 microscope.

### Quantitative Real time PCR

Total RNA from tissues was isolated by the acid guanidinium thiocyanate/phenol/chloroform method. Real time PCR was performed using the Prism 7000 Sequence Detection System (Applied Biosystems) with the SuperScript III Platinum SYBR Green One-Step qRT-PCR Kit (InVitrogen). The primer sequences for mouse SIRT1 are: forward 5′- AAT GCT GGC CTA ATA GAC TTG CA -3′, reverse 5′- CCG TGG AAT ATG TAA CGA TTT GG -3′. The primer sequences for mouse APE/Ref-1 are: forward 5′- -3′, reverse 5′- -3′. Mouse GAPDH was used as an internal control. The primer sequences for mouse GAPDH are: forward, 5′-GGC AAA TTC AAC GGC ACA GT-3′; reverse, 5′-CGC TCC TGG AAG ATG GTG AT-3′.

### Free Thiol Content of SIRT1 in Cells and Tissues

Reduced (free) sulfhydryl (thiol) residues in SIRT1 were detected by a modified biotin-switch assay [15] using biotin-HPDP as the reactant which covalently binds to reduced sulfhydryl groups. Nuclear extracts from cells and tissues were prepared in non-reducing HENT buffers (250 mM Hepes, pH 7.5 1 mM EDTA, 0.1 mM neocuproine, 1% Triton X-100). Typically, 1 mg of cell lysate and 2 mg of tissue homogenate was used. Free (reduced) thiols were linked with biotin using Biotin-HPDP (Santa Cruz Biotechnology). The biotinylated proteins were pulled down with Streptavidin-agarose. The pulled down proteins were immunoblotted with SIRT1 or myc antibody.

### Free Sulfhydryl Groups (Free Thiol Content) in Recombinant Proteins

cDNAs of wild-type and mutant SIRT1 and APE1/Ref -1 were cloned into pGEX expression vector. Proteins were expressed and induced with isopropyl β-d-thiogalactoside (IPTG) (0.1 mM) in BL21 (Stratagene) bacterial host strain. Expressed proteins were purified using glutathione Sepharose beads (Amersham Biosciences) following batch purification protocol recommended by the manufacturer. Eluted proteins were dialyzed to remove glutathione. Purity of the eluted fractions was determined by SDS/PAGE and Coomassie staining. A modified biotin switch approach [15] was used to measure the reduction of oxidized thiols in recombinant SIRT1 by recombinant APE1/Ref-1. Reduced sulfhydryls were first blocked with N-Ethylmaleimide (NEM) (Sigma). After removing free NEM with dialysis, recombinant proteins were incubated with biotin-HPDP, immobilized on Streptavidin-agarose beads and immunoblotted with SIRT1 antibody. Total SIRT1 and APE1/Ref-1 was detected using GST antibody.

### SIRT Activity Assay

The Biomol SIRT1 activity assay (AK-555, Biomol International) was used per manufacturer’s instructions to measure SIRT1 activity. Recombinant SIRT1 (SE239, Enzo) (with and without pre-incubation with wild-type APE1/Ref-1 or APE1/Ref-1 (C65A/C93A), or SIRT1 immunoprecipitated under non-reducing conditions from nuclear extracts of cells or tissues, was used. Fluorescence (Ex. 360 nm, Em. 460 nm) was measured with CytoFluor™ II, PerSeptive Biosystems. Activity was measured in the presence and absence of the SIRT1 inhibitor nicotinamide (NAM 5 mM), and difference in fluorescence units was calculated. Fluorescence units from immunoprecipitates using non-immune IgG was subtracted as background.

### Mouse Aortic Vascular Reactivity

8–12 week old APE1/Ref-1^+/+^ and APE1/Ref-1^+/−^ male mice were anesthetized and euthanized by rapid cardiac excision. The descending thoracic aorta was carefully excised and placed in ice-cold Krebs buffer (118.3 mM NaCl/4.7 mM KCl/2.5 mM CaCl_2_/1.2 mM KH_2_PO_4_/25 mM NaHCO_3_/1.2 mM MgSO_4_/11 mM glucose/0.0026 mM CaNa_2_EDTA). The aorta was cleaned of excess fat, cut transversely into 5–10 rings (2.0–3.0 mm), each of which was infected with 6×10^11^ viral particles per ml of the AdLacZ and AdSIRT1 adenoviral stocks, and incubated at 37°C for 24 h. The next day the vessels were placed in oxygenated chambers (95% O_2_/5% CO_2_) superfused with Krebs buffer solution and maintained at 37°C and pH 7.4. Each ring was suspended between two wire stirrups in a 5-ml organ chamber of a four-chamber myograph system (DMT). One stirrup was connected to a three-dimensional micromanipulator and the other to a force transducer. The contractile force was recorded electronically. All rings were stretched to 2,000 mg in 500-mg increments over a 1-h period to optimize the contractile response to KCl. One dose of KCl (60 mM) was administered to verify vascular smooth muscle viability. Cumulative dose–response curve for phenylephrine (10^−9^ to 10^−5^ M) was obtained by administering the drug in one-half log doses. Endothelium-dependent vasodilatation was determined by generating dose–response curves to acetylcholine. Vasorelaxation evoked by acetylcholine was expressed as percent contraction determined by the percentage of inhibition to the preconstricted tension. Endothelium-dependent NOS-independent vasorelaxation was assessed by generating dose–response curves to acetylcholine in rings pretreated with the NOS inhibitor L-NAME (10^−4^ M). NO bioavailability was measured physiologically by determining the increase in contractile response to inhibitor L-NAME in rings preconstricted with phenylephrine (10^−6^ M). Endothelium-independent vasodilatation was measured by the vasorelaxation evoked by cumulative sodium nitroprusside in rings preconstricted with phenylephrine (10^−6^ M).

### Ethics Statement

All animal experimentation was carried out under humane standards and was approved by the University of Pittsburgh Institutional Animal Care and Use Committee.

### Statistical Analysis

All experiments were performed at least three times. Data are expressed as mean ± SD. Statistical analysis was performed with SigmaStat. Data in which two conditions were compared were tested using the Student t-test. Data in which more than two conditions were compared in a single experiment were tested using ANOVA or repeated measures of ANOVA as appropriate. Correlation between variables was evaluated using the Pearson product method. A P-value of <0.05 was considered statistically significant.

## Results and Discussion

**Figure 1:**
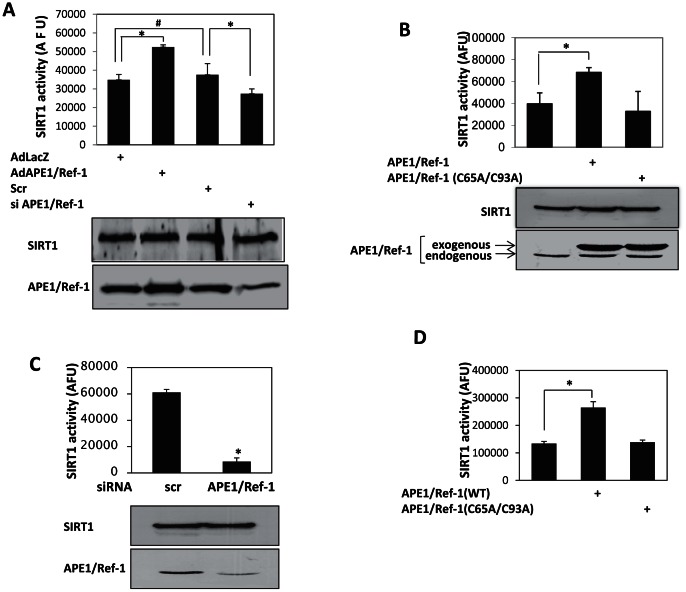
APE1/Ref-1 regulates SIRT1 activity in vitro and in vivo. (**A**) APE1/Ref-1 increases SIRT1 activity in endothelial cells. APE1/Ref1 was adenovirally over-expressed (AdAPE1/Ref-1) or knocked down with siRNA (siAPE1/Ref-1) in HUVEC. Inert adenovirus AdLacZ or scrambled siRNA (scr) were used as controls. Deacetylase activity of immunoprecipitated endogenous SIRT1 was measured. * *P*<0.05, # p>0.05, (n = 3). (**B**) Redox-deficient APE1/Ref-1 does not increase SIRT1 activity. HEK293 cells were transfected with APE1/Ref-1 (WT) or the redox-deficient APE1/Ref-1 (C65A/C93A) mutant. Deacetylase activity of immunoprecipitated endogenous SIRT1 was measured. * *P*<0.05, (n = 5). (**C**) Knockdown of APE1/Ref-1 decreases SIRT1 activity in HEK293 cells. Cells were transfected with APE1/Ref-1 siRNA or scrambled (scr) siRNA. Deacetylase activity of immunoprecipitated endogenous SIRT1 was measured. * *P*<0.05, (n = 5). (**D**) APE1/Ref-1 stimulates deacetylase activity of SIRT1 in vitro. Recombinant SIRT1 was incubated with recombinant wild-type (WT) or redox-deficient APE1/Ref-1 (C65A/C93A). Deacetylase activity was measured in the incubation mix. * *P*<0.05, (n = 5). In all experiments, deacetylase activity of recombinant SIRT1, or SIRT1 immunoprecipitated from cell lysates, was measured using the Fluor de Lys® Substrate and is expressed in arbitrary fluorescence units (AFU).

We first examined if APE1/Ref-1 affects SIRT1 activity in endothelial cells. APE1/Ref-1 was adenovirally overexpressed or knocked down with siRNA in HUVEC and the enzymatic activity of immunoprecipitated SIRT1 measured. Overexpression of APE1/Ref-1 stimulated endogenous SIRT1 activity, whereas knockdown of APE1/Ref-1 decreased this activity (Fig. 1A). In non-endothelial HEK 293 cells as well, overexpression of APE1/Ref-1 increased, while knockdown of APE1/Ref-1 decreased, endogenous SIRT1 activity (Fig. 1B, C).

We then asked if APE1/Ref-1 has a direct stimulatory effect on SIRT1. To do this, the activity of purified recombinant SIRT1 was measured in the presence of recombinant wild-type APE1/Ref-1. The activity of recombinant SIRT1 in vitro was increased in the presence of APE1/Ref-1 (Fig. 1D). Thus, in both cells and in vitro, APE1/Ref-1 stimulates SIRT1 activity, and lack of APE1/Ref-1 diminishes this activity.

APE1/Ref-1 is a multi-functional protein. One of its principal functions is reduction of nuclear transcription factors, maintaining them in the DNA-binding form. Cysteine residues at positions 65 and 93 in APE1/Ref-1 are essential for executing this function [16]. Hypothesizing that this reducing function of APE1/Ref-1 is responsible for APE1/Ref-1-stimulated SIRT1 activity, we examined the effect APE1/Ref-1 mutated at cysteines 65 and 93, which is devoid of this reducing function, on SIRT1activity. In contrast to wild-type APE1/Ref-1, the redox-deficient mutant of APE1/Ref-1 (C65A/C93A) did not stimulate SIRT1 activity in HEK 293 cells (Fig. 1B) or in vitro (Fig. 1D). Thus APE1/Ref-1 stimulates SIRT1 activity by virtue of its reducing function.

**Figure 2:**
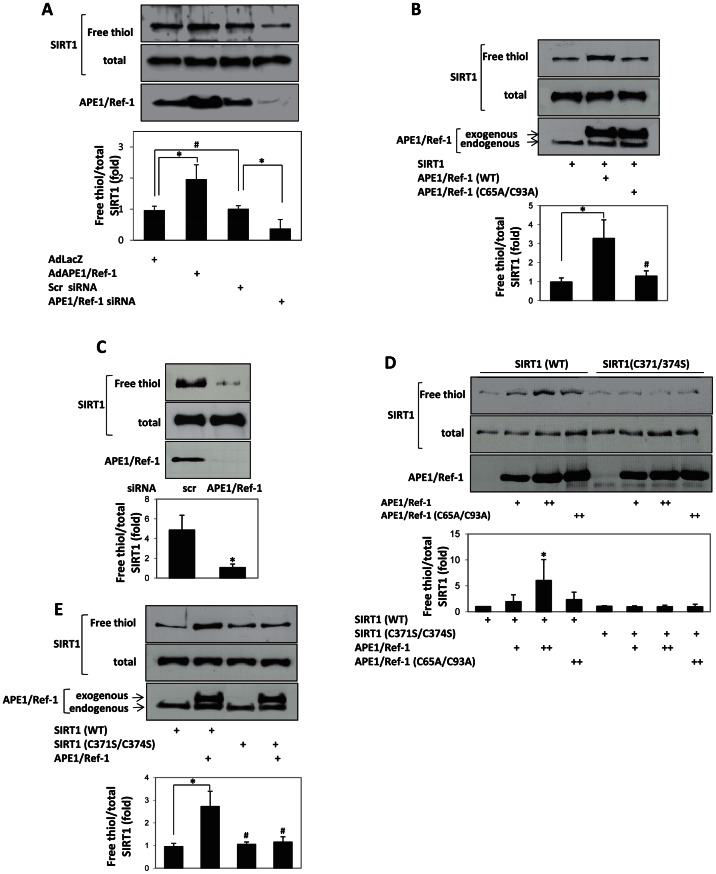
APE1/Ref-1 increases free thiol content of SIRT1. (**A**) APE1/Ref-1 reduces thiol residues of SIRT1 in endothelial cells. APE1/Ref1 was adenovirally over-expressed (AdAPE1/Ref-1) or knocked down with siRNA (siAPE1/Ref-1) in HUVEC. Inert adenovirus AdLacZ or scrambled siRNA (scr) were used as controls. * *P*<0.05, (n = 3). (**B**) Redox-deficient APE1/Ref-1 does not reduce thiol residues of SIRT1 or stimulate SIRT1 activity. HEK293 cells were transfected with APE1/Ref-1 (WT) or the redox-deficient APE1/Ref-1 (C65A/C93A) mutant. * *P*<0.05, # p>0.05, (n = 3). (**C**) Knockdown of APE1/Ref-1 decreases free thiol content of SIRT1 in HEK293 cells. Cells over-expressing SIRT1 were transfected with APE1/Ref-1 siRNA or scrambled (scr) siRNA. * *P*<0.05, (n = 3). (**D**) Cysteines 371 and 374 in the catalytic core domain of SIRT1 are targeted for reduction by APE1/Ref-1. Recombinant wild-type SIRT1 (WT) and SIRT1 mutated at cysteines 371 and 374 (C371S/C374S) were incubated with wild-type APE1/Ref-1 (WT) or redox-deficient APE1/Ref-1 (C65A/C93A) in vitro. Reduced cysteines in SIRT1 and total SIRT1 protein were densitometrically quantified. * *P*<0.05, (n = 3) (**E**) APE1/Ref-1 does not reduce thiol residues in SIRT1 mutated at cysteines 371 and 374 in HEK 293 cells. Wild-type (WT) SIRT1 and SIRT1 (C371S/374S), with and without APE1/Ref-1, were expressed in cells. In all experiments, free thiol content of SIRT1 was determined using a modified biotin-switch assay, and is expressed after normalization with total SIRT1.

We next examined if APE1/Ref-1 changes the quantity of reduced cysteine sulfhydryls (free thiol content) of endothelial SIRT1. APE1/Ref-1 was adenovirally overexpressed or knocked down with siRNA in HUVEC and free thiol content of immunoprecipitated SIRT1 assessed. Overexpression of APE1/Ref-1 increased free thiols in endogenous SIRT1, whereas knockdown of APE1/Ref-1 decreased free thiol content of endothelial SIRT1 (Fig. 2A). In non-endothelial HEK 293 cells as well, overexpression of APE1/Ref-1 increased, while knockdown of APE1/Ref-1 decreased, free thiols in SIRT1 (Fig. 2B, C).

We then determined if APE1/Ref-1 increases free thiols of SIRT1 through direct reduction of these residues. To do this, we examined the effect of purified APE1/Ref-1 on free thiol content of recombinant SIRT1 in vitro. APE1/Ref-1 dose-dependently increased the free thiol content of recombinant SIRT1 (Fig. 2D). In contrast, purified redox-deficient APE1/Ref-1 (C65A/C93A) did not increase free thiol content of SIRT1 (Fig. 2D). Thus, APE1/Ref-1, via its redox function, directly reduces cysteine sulfhydryls in SIRT1.

The deacetylase domain of SIRT1/Sir2 has a zinc tetra-thiolate center which is highly conserved from yeast to mammals. We asked if the cysteine residues in SIRT1 which are reduced by APE1/Ref-1 are in this tetra-thiolate motif. To answer this we created a mutant of SIRT1 in which two cysteines in this tetra-thiolate motif are mutated to non-reducible serines (C371S/374S). The effect of APE1/Ref-1 on free thiol content of SIRT1 (C371S/374S) in vitro and in cells was then examined. In contrast to wild-type recombinant SIRT1, the free thiol content of recombinant SIRT1 (C371S/374S) was not increased by purified APE1/Ref-1 (Fig. 2D). Similarly, in HEK 293 cells, APE1/Ref-1 did not increase free thiol content of SIRT1 (C371S/374S) (Fig. 2E). Thus, APE1/Ref-1 targets cysteines 371 and 374, which form the Zn tetra-thiolate motif in the deacetylase domain of SIRT1, for reduction.

**Figure 3:**
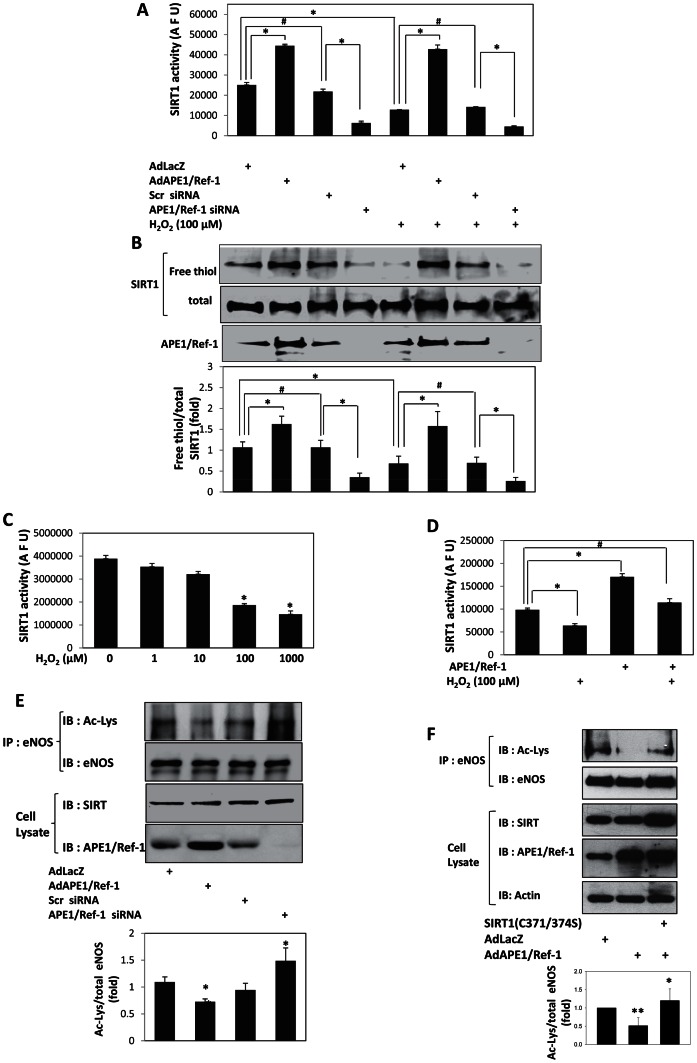
APE1/ref-1 protects endothelial SIRT1 from oxidative stress-induced inactivation and promotes de-acetylation of endothelial nitric oxide synthase. (**A**) H_2_O_2_-induced decrease in SIRT1 activity is rescued by APE1/Ref-1. APE1/Ref1 was adenovirally over-expressed (AdAPE1/Ref-1) or knocked down with siRNA (siAPE1/Ref-1) in HUVEC. Inert adenovirus AdLacZ or scrambled siRNA (scr) were used as controls. Cells were treated with H_2_O_2_ for 30 min. * *P*<0.05, # p>0.05, (n = 3). (**B**) H_2_O_2_-induced decrease in free thiol content of SIRT1 is rescued by APE1/Ref-1. APE1/Ref1 was adenovirally over-expressed (AdAPE1/Ref-1) or knocked down with siRNA (siAPE1/Ref-1) in HUVEC. Inert adenovirus AdLacZ or scrambled siRNA (scr) were used as controls. Cells were treated with H_2_O_2_ for 3 hrs. * *P*<0.05, # p>0.05, (n = 3). (**C**) H_2_O_2_ dose-dependently inactivates SIRT1 in vitro. Recombinant active SIRT1 was treated with H_2_O_2_ for 1 hour. * *P*<0.05, (n = 3) In A-C, deacetylase activity of recombinant SIRT1, or SIRT1 immunoprecipitated from cell lysates, was measured using the Fluor de Lys® Substrate and is expressed in arbitrary fluorescence units (AFU). Free thiol content of SIRT1 was determined using a modified biotin-switch assay, and is expressed after normalization with total SIRT1. (**D**) APE1/Ref-1 rescues SIRT1 from H_2_O_2_–induced inactivation in vitro. Recombinant active SIRT1 was treated with H_2_O_2_ for 1 hour. * *P*<0.05, # p>0.05, (n = 3). (**E**) APE1/Ref-1 stimulates lysine de-acetylation of endothelial nitric oxide synthase (eNOS). APE1/Ref1 was adenovirally over-expressed (AdAPE1/Ref-1) or knocked down with siRNA (siAPE1/Ref-1) in HUVEC. Inert adenovirus AdLacZ or scrambled siRNA (scr) were used as controls. Acetylated lysine was assessed and quantified in immunoprecipitated eNOS. Values are expressed as fold change in acetylated eNOS after normalization with total eNOS. * *P*<0.05, # p>0.05, (n = 3). (**F**) Non-reducible SIRT1 inhibits APE1/Ref-1-stimulated deacetylation of eNOS. APE1/Ref-1, with or without non-reducible SIRT1 (C371S/C374S) was expressed in HUVEC. Acetylated eNOS was assessed in acetyl-lysine immunoprecipitates. * *P*<0.05 compared to control, and ** *P*<0.01 compared to APE1/Ref-1+ SIRT1 (C371S/C374S), (n = 3).

SIRT1 is prone to redox modifications such as glutathiolation [4]. Therefore, we asked if the reactive oxygen moiety hydrogen peroxide can post-translationally modify SIRT1 and affect its activity in endothelial cells. The activity and free thiol content of endothelialSIRT1was significantly diminished by hydrogen peroxide (Fig. 3A, B). Hydrogen peroxide also inhibited the activity of recombinant SIRT1 in vitro (Fig. 3C). Thus, hydrogen peroxide oxidizes cysteine sulfhydryls in endothelial SIRT1, thereby inhibiting its activity.

APE1/Ref-1 protects endothelial cells from the deleterious consequences of oxidative stress [17]. Therefore, we asked if APE1/Ref-1 also protects endothelial SIRT1 for hydrogen peroxide-induced inactivation. Overexpression of APE1/Ref-1 in endothelial cells mitigated hydrogen peroxide-induced decrease in free thiol content and deacetylase activity of SIRT1 (Fig. 3A, B). Conversely, knockdown of APE1/Ref-1 in endothelial cells led to a decrease in free thiol content and activity of SIRT1 (Fig. 3A, B). Purified APE1/Ref-1 also rescued inactivation of recombinant SIRT1 by hydrogen peroxide in vitro (Fig. 3D). Thus, endogenous APE1/Ref-1 is obligatory for optimal endothelial SIRT1 activity, and overexpression of APE1/Ref-1 protects endothelial SIRT1 from inactivation by oxidative stress.

APE1/Ref-1 stimulates the activity of eNOS and increases endothelial NO [13]. Endothelial nitric oxide synthase (eNOS) is lysine acetylated and SIRT1 increases eNOS activity by deacetylating it [18]. We therefore wondered if the effect of APE1/Ref-1 on endothelial SIRT1 translates into a change in eNOS acetylation. In endothelial cells, overexpression of APE1/Ref-1 suppressed lysine acetylation of eNOS, whereas knockdown of APE1/Ref-1 increased eNOS acetylation (Fig. 3E). This suggests that activation of endothelial SIRT1 by APE1/Ref-1 translates into deacetylation of eNOS.

Next, we asked if reduction of SIRT1 is required for deacetylation of eNOS by APE1/Ref-1. We first measured deacetylase activity of non-reducible SIRT1 (C371S/C374S). Compared to WT SIRT1, deacetylase activity of SIRT1 (C371S/C374S) was significantly diminished in both HUVEC and HEK 293 cells (Fig. S1A, and S1B). Moreover, expression of SIRT1 (C371S/C374S) inhibited the activity of endogenous SIRT1 (Fig. S1C) indicating that it acts in a dominant inhibitory manner. Capitalizing on this property of SIRT1 (C371S/C374S), we determined if it can negate APE1/Ref-1-induced deacetylation of eNOS. In HUVEC, expression of SIRT1 (C371S/C374S) prevented deacetylation of eNOS by APE1/Ref-1 (Fig. 3F) indicating that reduction of these cysteine sulfhydryls in SIRT1by APE1/Ref-1 is obligatory for APE1/Ref-1-induced deacetylation of eNOS.

**Figure 4:**
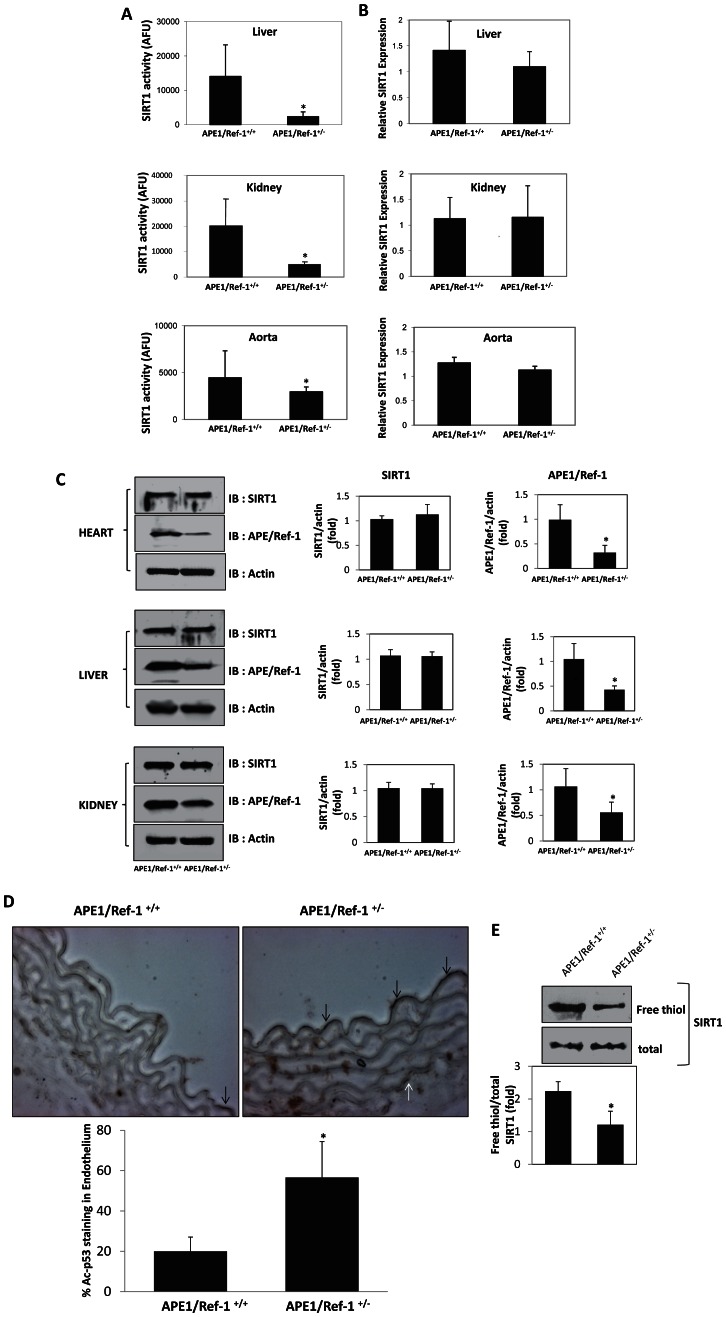
SIRT1 activity and free thiol content is diminished in APE1/Ref-1 heterozygous mice. (**A**) APE1/Ref-1^+/−^ mice have decreased tissue SIRT1 activity. SIRT1 activity was measured in liver, kidney and aorta of APE1/Ref-1^+/−^ mice and APE1/Ref-1^+/+^ mice. * *P*<0.05, (n = 3). (**B and C**) SIRT1 expression is unchanged in APE1/Ref-1^+/−^ mice. SIRT1 mRNA (B) and protein (C), and APE1/Ref-1 protein (C) was assessed and quantified in liver, kidney, heart and aorta of APE1/Ref-1^+/+^ mice and APE1/Ref-1^+/−^ mice. mRNA was normalized to GAPDH, and protein to β-actin, and both expressed as fold relative to APE1/Ref-1^+/+^ mice. * *P*<0.05, # p>0.05, (n = 3). (**D**) Free thiol content of tissue SIRT1 is diminished in APE1/Ref-1^+/−^ mice. Free thiol content was assessed and densitometrically quantified in kidneys of APE1/Ref-1^+/+^ and APE1/Ref-1^+/−^ mice. * *P*<0.05, (n = 3). (**E**) APE1/Ref-1^+/−^ mice have increased lysine acetylation of endothelial p53. Immunohistochemistry for lysine acetylated p53 was performed in sections of aortas from APE1/Ref-1^+/+^ and APE1/Ref-1^+/−^ mice. Magnification is 40×. Percentage of endothelial cells positive for detectable lysine acetylated p53 was quantified in 20 cells from three separate fields. * *P*<0.05, (n = 3). Arrows: positive cells identified in the endothelium and media. Deacetylase activity of SIRT1 immunoprecipitated from nuclear extracts of homogenized tissues was measured using the Fluor de Lys® Substrate and is expressed in arbitrary fluorescence units (AFU). Free thiol content of SIRT1 in nuclear extracts was determined using a modified biotin-switch assay, and is expressed after normalization with total SIRT1.

We then explored the in vivo significance of the afore-mentioned relationship between APE1/Ref-1 and SIRT1 using mice heterozygous for APE1/Ref-1. Compared with wild-type littermates, aortic tissue of APE1/Ref-1^+/−^ mice had lower SIRT1 activity (Fig. 4A). Decreased SIRT1 activity was not restricted to the vasculature, as all tissues examined in APE1/Ref-1^+/−^ mice had lower SIRT1 activity than wild-type controls (Fig. 4A). Diminished tissue SIRT1 activity was not due to down-regulation of SIRT1, as expression of SIRT1, both at the mRNA and protein levels, was similar in wild-type and APE1/Ref-1^+/−^ mice (Fig. 4B, C). In addition to lower SIRT1 activity in whole aortas, endothelial SIRT1 activity was also diminished in APE1/Ref-1^+/−^ mice, as reflected by an increase in lysine acetylated p53, a protein that SIRT1 targets for deacetylation (Fig. 4D). Decreased SIRT1 activity mirrored diminished free thiol content of SIRT1 in tissues of APE1/Ref-1^+/−^ mice (Fig. 4E). Therefore, lack of one allele of APE1/Ref-1 in mice leads to diminished deacetylase activity and decreased free thiol content of tissue SIRT1.

**Figure 5:**
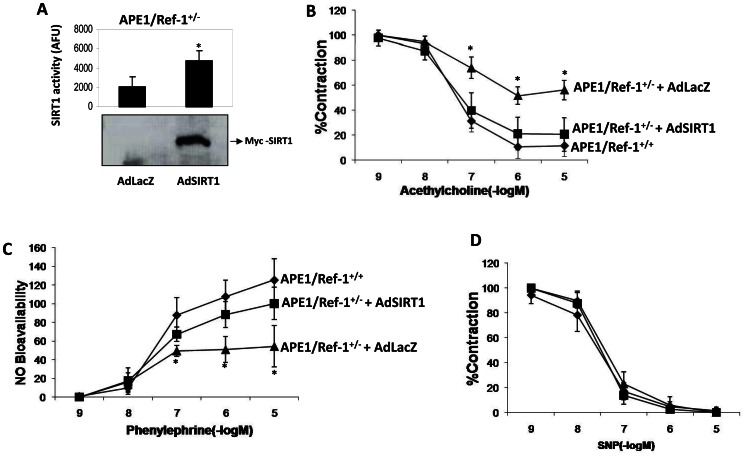
SIRT1 rescues impaired endothelium-dependent vasorelaxation in APE1/Ref-1 heterozygous mice. (**A**) Adenoviral gene transfer of SIRT1 increases vascular SIRT1 activity. Wild-type SIRT1 was overexpressed ex vivo in aortic sections of APE1/Ref-1^+/−^ mice using AdSIRT1. AdLacZ was used as control. SIRT1 protein and activity in whole aortas was determined. * *P*<0.05, (n = 3) (**B**) Overexpression of SIRT1 restores endothelium-dependent vasorelaxation in APE1/Ref-1^+/−^ mice. Wild-type SIRT1 was overexpressed ex vivo in aortic sections of APE1/Ref-1^+/−^ mice using AdSIRT1. AdLacZ was used as control. Acetylcholine-induced endothelium-dependent vasorelaxation was measured in aortic sections of APE1/Ref-1^+/+^ mice (♦), APE1/Ref-1^+/−^ mice infected with AdSIRT1 (▪), and APE1/Ref-1^+/−^ mice infected with AdLacZ (▴).* *P*<0.01 compared with APE1/Ref-1^+/+^ and APE1/Ref-1^+/−^+AdSIRT1 (n = 6). (**C**) Bioavailable NO in aortic sections of APE1/Ref-1^+/+^ mice (♦), APE1/Ref-1^+/−^ mice infected with AdSIRT1 (▪), and APE1/Ref-1^+/−^ mice infected with AdLacZ (▴).*P<0.01 compared with APE1/Ref-1^+/+^ mice and APE1/Ref-1^+/−^ mice**+**AdSIRT1 (n = 5). (**D**) Sodium nitroprusside (SNP)-induced endothelium-independent vasorelaxation in aortic sections of APE1/Ref-1^+/+^ mice (♦), APE1/Ref-1^+/−^ mice infected with AdSIRT1 (▪), and APE1/Ref-1^+/−^ mice infected with AdLacZ (▴) (n = 4).

APE1/Ref-1^+/−^ mice have impaired endothelium-dependent vasorelaxation, lower vascular nitric oxide, and are hypertensive [13]. Similar to the role of APE1/Ref-1 in the endothelium, SIRT1 promotes vascular nitric oxide availability and endothelium-dependent vasorelaxation [18], inhibits endothelial activation [19], decreases high-fat diet-induced atherosclerosis [20], and mediates shear stress-stimulated vascular NO production [21]. Hypothesizing that impaired endothelial vasodilatory function in APE1/Ref-1^+/−^ mice may, in part, be due to diminished vascular SIRT1 activity, we asked if SIRT1 rescues endothelium-dependent vascular dysfunction in these mice. SIRT1 was adenovirally overexpressed (AdSIRT1) in aortas of APE1/Ref-1^+/−^ mice. The adenovirus AdLacZ, expressing the inert *E. Coli* β-galactosidase gene, was used as a control. AdSIRT1 increased SIRT1 activity in aortas of APE1/Ref-1^+/−^ mice (Fig. 5A). Increase in SIRT1 activity restored acetylcholine-induced endothelium-dependent vasorelaxation in APE1/Ref-1^+/−^ mice to that of wild-type animals (Fig. 5B). In addition, adenoviral overexpression of SIRT1 restored bioavailable NO in aortas of APE1/Ref-1^+/−^ mice to levels comparable to those of wild-type mice (Fig. 5C). Endothelium-independent vasorelaxation elicited by the NO donor sodium nitroprusside (SNP) was similar in the wild-type and APE1/Ref-1^+/−^ mice and was not affected by SIRT1 overexpression (Fig. 5D). Thus, SIRT1 overexpression rescues impaired endothelium-dependent vasorelaxation due to deficiency of APE1/Ref-1.

Identification of SIRT1 as a target of APE1/Ref-1 adds to a substantial number of proteins reduced by APE1/Ref-1. Many of these proteins are transcription factors, and their reduction by APE1/Ref-1 facilitates their binding to DNA or transcriptional co-activators. Interestingly, some of these same transcription factors have been identified as targets for deacetylation by SIRT1 [22], [23]. This raises the possibility that APE1/Ref-1 and SIRT1 may act in concert to modulate transcription by these factors. For example, APE1/Ref-1 reduces cysteines in the DNA-binding domain of c-jun, promoting binding of the fos-jun complex to DNA, and stimulating transcriptional activity of activator protein-1 (AP-1) [24], [25]. SIRT1, on the other hand, deacetylates c-fos and c-jun, inhibiting AP-1 transcriptional activity [23]. Thus, reductive activation of SIRT1 by APE1/ref-1 may be part of a feedback loop modulating the activation of the AP-1 transcription complex. A similar scenario can be envisioned for other transcription factors such as the tumor suppressor protein p53, and hypoxia-inducible factor-1α, both of which are targeted by SIRT1 and APE1/Ref-1 [22], [26], [27], [28].

**Figure 6:**
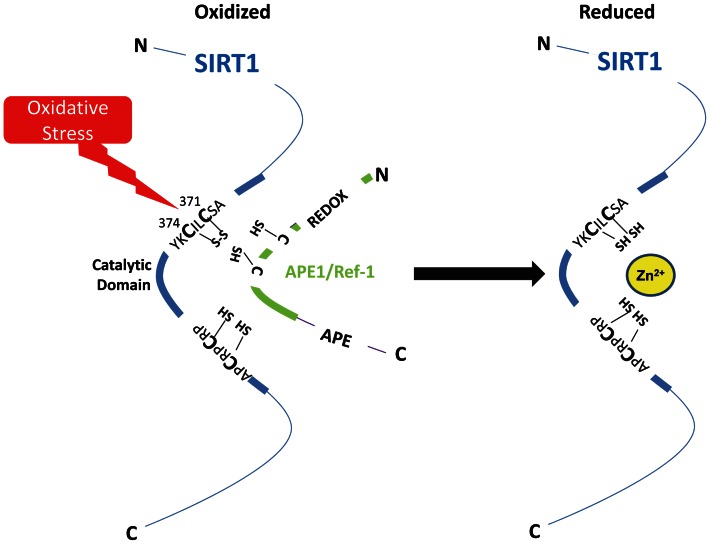
Schematic showing oxidation of vicinal thiols in the zinc tetra-thiolate center of the catalytic domain of SIRT1, and their reduction by APE1/Ref-1.

The four cysteine residues in the zinc tetra-thiolate center of SIRT1 are part of two CXXC (where X is any amino acid) motifs. All four vicinal thiols in these two motifs are highly conserved, from yeast Sir2 to mammalian SIRT1 [7]. Two of these thiols are S-nitrosylated by nitric oxide [5]. Our work examined the effect of APE1/Ref-1 on the redox status of the other two cysteine residues in this tetra-thiolate center. Although we did not test whether the two cysteines subject to modification by NO are also subject to reduction by APE1/Ref-1, it would not be surprising if that were the case, given that mutational disruption of any one of the two CXXC motifs in yeast Sir2 renders the enzyme inactive [8]. Importantly, the vicinal nature of cysteines 371 and 374 lends these residues to intra-molecular disulfide bond formation by endogenous oxidants and oxidative stress (Fig. 6), whereas other cysteines in SIRT1, such as cysteines 67, 268, and 623 which are also susceptible to post-translational modifications [4], [6], not being vicinal thiols, may not be able to form disulfide bonds in oxidizing conditions. This could explain why cysteines 371 and 374 were the residues targeted by APE1/Ref-1 (Fig. 2D, E). However, the inference that APE1/Ref-1 targets only vicinal thiols in SIRT1 must be tempered by the realization that it can also reduce non-vicinal cysteine residues in many of its target proteins.

Endothelial SIRT1 has a vital part in choreographing pre- and post-natal angiogenesis [29]. The redox function of APE1/Ref-1 too, has an important role in neo-vascularization, as evidenced using selective inhibitors of APE1/Ref-1 in models of retinal angiogenesis [30]. Given our observations about the importance of APE1/Ref-1 to endothelial SIRT1 activity, it is tempting to speculate that redox regulation of SIRT1 by APE1/Ref-1, and possibly by other cellular reducing equivalents, not only mediates vasodilation, but may also be relevant to other vital endothelial functions such as angiogenesis.

## Supporting Information

Figure S1 **SIRT1 (C371S/C374S) has diminished deacetylase activity and acts in a dominant negative fashion. (A, B)** SIRT1 (C371S/C374S) has diminished deacetylase activity. Deacetylase activity of immunoprecipitated SIRT1 (WT) and SIRT1 (C371S/C374S) expressed in (**A**) HUVEC and (**B**) HEK 293 cells was determined. Values are expressed relative to SIRT1 (WT). *, ***P<0.05, 0.001 (n = 3–4). **(C)** SIRT1 (C371S/C374S) inhibits the activity of endogenous SIRT1. Deacetylase activity of immunoprecipitated total SIRT1 was measured in HEK 293 cells not expressing ectopic SIRT1 and those expressing SIRT1 (C371S/C374S). *** *P*<0.001 (n = 3). Deacetylase activity of SIRT1 immunoprecipitated from cell lysates was measured using the Fluor de Lys® Substrate.
(TIF)
