# Supplementary figures and images for: Redox Factor-1 Activates Endothelial SIRTUIN1 through Reduction of Conserved Cysteine Sulfhydryls in Its Deacetylase Domain
Source: PLoS One. 2013 Jun 3;8(6):e65415. doi: 10.1371/journal.pone.0065415 (PMC3670896; doi:10.1371/journal.pone.0065415)

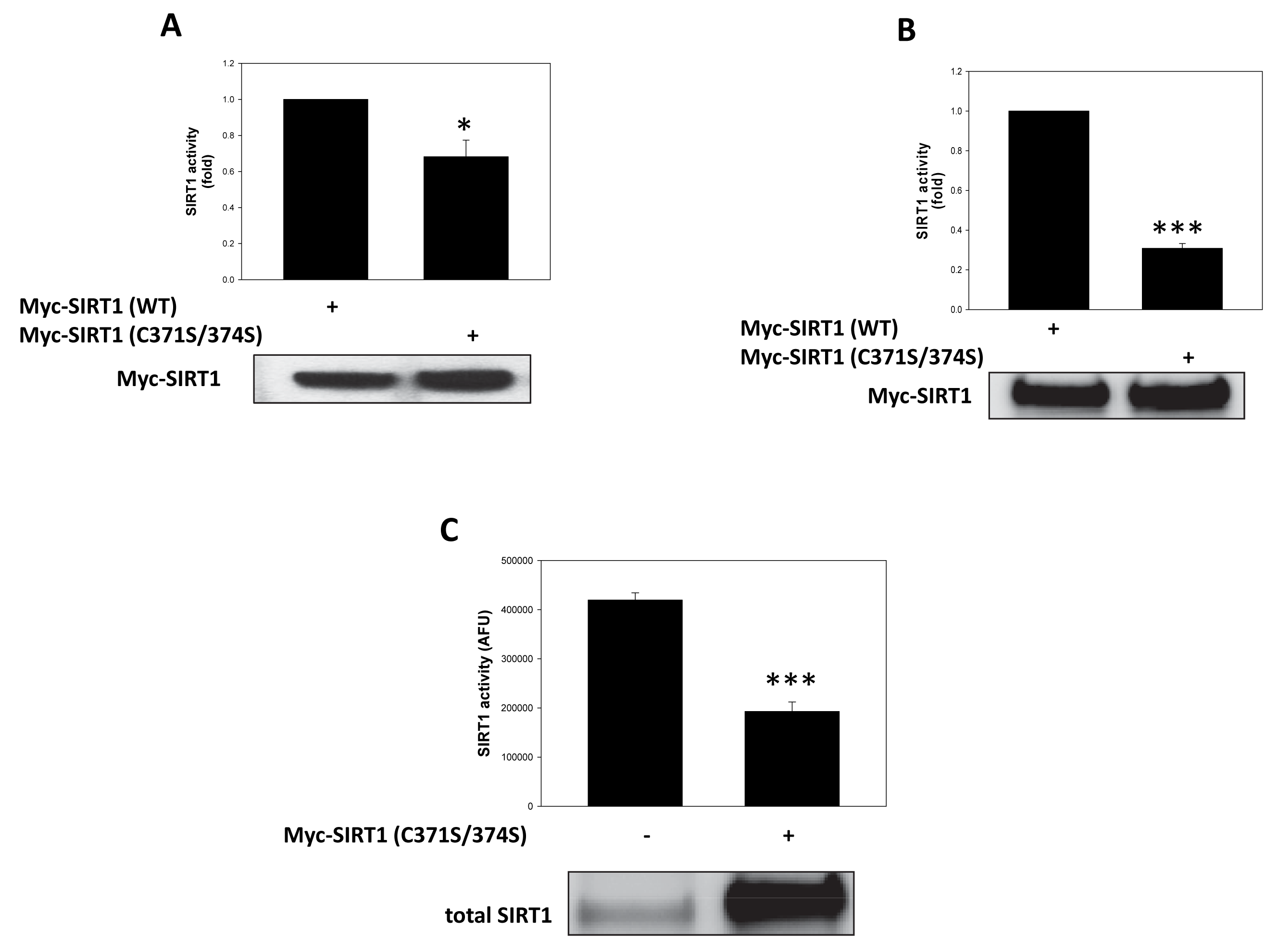

Supplement: Figure S1 — SIRT1 (C371S/C374S) has diminished deacetylase activity and acts in a dominant negative fashion. (A, B) SIRT1 (C371S/C374S) has diminished deacetylase activity. Deacetylase activity of immunoprecipitated SIRT1 (WT) and SIRT1 (C371S/C374S) expressed in (A) HUVEC and (B) HEK 293 cells was determined. Values are expressed relative to SIRT1 (WT). *, ***P<0.05, 0.001 (n = 3–4). (C) SIRT1 (C371S/C374S) inhibits the activity of endogenous SIRT1. Deacetylase activity of immunoprecipitated total SIRT1 was measured in HEK 293 cells not expressing ectopic SIRT1 and those expressing SIRT1 (C371S/C374S). *** P<0.001 (n = 3). Deacetylase activity of SIRT1 immunoprecipitated from cell lysates was measured using the Fluor de Lys® Substrate. (TIF) [file pone.0065415.s001.tif]
